# Nutritional Evaluation of Traditional Portuguese Recipes from Three Different Regions: A Contribution to Food Composition Databases

**DOI:** 10.3390/nu18152486

**Published:** 2026-08-01

**Authors:** Mariana Lourenço, Paula Pereira, Marta Neves, Manuel Bicho, Ana Valente

**Affiliations:** 1 Applied Nutrition Research Group (GENA), Nutrition Lab, Egas Moniz Center for Interdisciplinary Research (CiiEM), Egas Moniz School of Health & Science, 2829-511 Caparica, Portugal; marianapl2002@gmail.com (M.L.); pmpereira@egasmoniz.edu.pt (P.P.); 2 Nutrialma^®^—Consultoria em Nutrição, 1250-008 Lisboa, Portugal; marta.neves@nutrialma-consulting.com; 3 Ecogenetics and Human Health Research Group, ISAMB—Institute of Environmental Health, Associate Laboratory TERRA, Lisbon School of Medicine, University of Lisbon, 1649-028 Lisboa, Portugal; manuelbicho@medicina.ulisboa.pt; 4 Institute of Scientific Research Bento da Rocha Cabral, Calçada Bento da Rocha Cabral 14, 1250-012 Lisbon, Portugal

**Keywords:** gastronomy, tradition, culture, public health, Portugal, nutritional composition, dietary guidelines

## Abstract

Background/Aim: Traditional Portuguese cuisine is a key component of the country’s cultural identity. Understanding how traditional Portuguese dishes align with, or diverge from, the principles of a balanced and sustainable diet is essential for promoting healthy eating habits while preserving cultural heritage. The aims of this study were: (a) to evaluate and compare the nutritional composition of traditional Portuguese recipes from different regions; and (b) to assess the adequacy of complete regional menus in relation to recommendations for a balanced and healthy meal. Methodology: A pilot study was conducted analysing 60 traditional Portuguese recipes from three regions of mainland Portugal (North, Centre, and South). Twenty recipes per region were evaluated, including six first courses, eight main courses, and six desserts. Nutritional composition was determined per 100 g, and regional differences were analysed using one-way ANOVA with Tukey’s post hoc test (*p* < 0.05). Six complete menus were also constructed and assessed against reference values for energy and nutrient intake. Results: Significant differences were observed in the energy (*p* = 0.02) and sodium (*p* = 0.03) content of first-course dishes, particularly between the Northern region and the other regions. The Central region showed higher mean dietary fibre values. No statistically significant regional differences were identified for main courses or desserts. Menu analysis revealed that most traditional menu combinations exceeded recommended energy values and showed imbalances in macronutrient distribution, notably excessive saturated fat and insufficient dietary fiber. Conclusions: Traditional Portuguese first-course dishes display relevant regional nutritional differences. Overall, complete traditional menus frequently fall short of current nutritional recommendations, emphasizing the need for culturally sensitive adaptations that maintain culinary heritage while supporting public health goals, namely by lowering saturated fat and sodium content and enhancing dietary fibre levels.

## 1. Introduction

Food is one of the most distinctive expressions of a people’s culture. In Portugal, eating and cooking habits reflect centuries of history, adaptation to the territory, and the transmission of knowledge across generations. Dietary practices have evolved according to natural resources, climatic conditions, social dynamics and regional economy, playing a central role in shaping collective identity [1]. More than a collection of recipes, Portuguese gastronomy represents a way of living and sharing, strongly associated with family interaction and a sense of community. From North to South, the country’s geographical and cultural diversity is reflected in its regional culinary traditions. In the North, dishes tend to be richer and more substantial, often associated with pastoral activities and harsher climatic conditions. In the Centre, inland and coastal influences coexist, with legumes, fish, and cereals playing a prominent role. In the South, characterised by higher temperatures and a clear Mediterranean heritage, lighter preparations based on olive oil, vegetables, and fish prevail [2]. This diversity of food practices reveals the close relationship between territory, local economy, and gastronomic preferences, constituting an intangible cultural heritage of high value. The history of Portuguese gastronomy is also the result of multiple external influences. Cultural exchanges, maritime discoveries, and commercial contacts introduced new ingredients and culinary techniques that enriched national cuisine [3]. The combination of local products with foreign influences contributed to the development of a diverse gastronomy in which simplicity and complexity, as well as tradition and innovation, coexist. Over time, this heritage has been recorded and preserved in reference compilations, among which *Cozinha Tradicional Portuguesa* by Maria de Lourdes Modesto (1981) [4] stands out as one of the most influential works in the transmission and valorisation of traditional recipes. Despite the cultural relevance of Portuguese gastronomy, its study from a nutritional perspective remains limited. This study contributes to strengthening scientific knowledge on traditional Portuguese gastronomy by valuing its cultural dimension through a nutrition-based analytical perspective. Most culinary literature focuses on describing preparation methods rather than analysing nutritional composition, making it difficult to assess the contribution of traditional recipes to contemporary dietary patterns. Furthermore, comparative nutritional evidence across the main Portuguese regions remains limited, making it difficult to identify regional nutritional patterns and opportunities for evidence-based recipe improvement while preserving culinary heritage. In a context where the promotion of balanced eating patterns is a public health priority, the scientific analysis of traditional recipes is essential to better understand their nutritional value and their role in modern diets. According to the Food and Agriculture Organization of the United Nations [5], traditional diets represent sustainable food systems developed through the interaction between the environment, local resources, and cultural practices. The Portuguese dietary pattern aligns with this concept, reflecting adaptation to territory and seasonality, while also incorporating practices that may diverge from current nutritional recommendations. In scientific literature, traditional food is defined as a product that has been consumed in the national market for a sufficiently long period to allow intergenerational transmission (≥30 years) [6]. Traditional recipes are therefore deeply rooted in the cultural heritage of each region and its people. Studying their nutritional composition is essential to highlight their potential as balanced dietary models and to identify aspects that may require improvement. Recent scientific research has reinforced interest in dietary approaches that integrate tradition with nutritional evidence. Studies by Dinu et al. (2018) [7] and Finicelli et al. (2022) [8] indicate that dietary patterns based on local products, vegetables, legumes, and olive oil are associated with the prevention of chronic diseases and the promotion of more sustainable eating habits. In this context, understanding how traditional Portuguese gastronomy aligns with these principles is relevant not only from a scientific perspective, but also from cultural and educational standpoints. Non-communicable chronic diseases, such as obesity and cardiovascular diseases, remain a major public health concern in Portugal [9], with diet and lifestyle recognized as the principal modifiable risk factors underlying their development. In this context, evaluating the nutritional balance of traditional recipes becomes particularly relevant, as these foods contribute to habitual dietary patterns. Assessing their nutritional profiles is therefore essential to promote a culturally rooted yet nutritionally adequate diet, supporting the design of effective and culturally grounded dietary interventions.

Therefore, a comparative study of the nutritional composition of 60 traditional Portuguese recipes, selected to represent the gastronomic diversity of the North, Centre, and South regions of mainland Portugal, was conducted to evaluate their contribution to population energy and nutrient intake. This analysis aimed to identify regional consumption patterns, potential nutritional excesses or deficiencies, and opportunities for recipe reformulation that reconcile the preservation of national gastronomic heritage with current public health and nutrition recommendations. The aim of this study was to provide an in-depth understanding of regional differences in Portuguese gastronomy from a nutritional perspective. The specific objectives were: (a) to evaluate and compare the nutritional composition of traditional Portuguese recipes representative of three regions of Portugal; and (b) to assess the adequacy of complete regional menus in relation to recommendations for a balanced and healthy meal. Given the exploratory nature of this pilot study, the selection of traditional recipes aimed to capture culturally representative dishes from each region rather than to ensure statistical representativeness. Nevertheless, this approach allows an initial characterisation of regional nutritional patterns and provides data that may support the future expansion and refinement of national food composition databases, particularly for composite traditional dishes.

## 2. Materials and Methods

### 2.1. Study Design

A pilot study was conducted to evaluate and compare the nutritional composition of traditional Portuguese recipes representative of the three main regions of mainland Portugal (North, Centre, and South), aiming to identify potential regional differences in their nutritional profiles. Recipes from these three major administrative regions defined by the National Institute of Statistics [10] were included in the analysis. Regional delimitation followed the Nomenclature of Territorial Units for Statistics (NUTS II) system, ensuring consistency in geographical classification and balanced representation among the regions analysed.

### 2.2. Data Collection

#### 2.2.1. Recipe Source Selection

The sample consisted of 60 traditional Portuguese recipes, evenly distributed across the three regions (20 recipes per region). Within each region, the selection included six first courses (two of which were soups), eight main courses (four meat-based dishes and four fish-based dishes), and six desserts. Recipe selection was based on two widely recognised reference sources, chosen for their authenticity, historical relevance, and cultural significance in Portuguese culinary literature:

- Cozinha Tradicional Portuguesa by Maria de Lourdes Modesto (1981) [4], widely regarded as the “Bible” of Portuguese gastronomy.
- Teleculinária magazine (2024) [11], first published in 1976, which became a benchmark in Portuguese cuisine and is currently available exclusively in digital format.

Recipes were selected according to the geographical origin indicated in the culinary publications, as well as their cultural representativeness and popularity within each region. Priority was given to preparations most strongly associated with local gastronomic identity and still present in contemporary Portuguese cuisine. Cultural representativeness was further validated using the VisitPortugal portal (Turismo de Portugal, 2024) [12] and the Portugal Gastronomias website [13], which identify emblematic gastronomic specialties of each region. The combined use of these sources ensured the cultural and regional adequacy of the sample, ensuring that the selected recipes accurately reflected Portuguese gastronomic heritage. Each recipe was subsequently coded using a numerical system corresponding to region and dish category, allowing systematic organisation and facilitating subsequent statistical analysis (Table 1).

#### 2.2.2. Inclusion/Exclusion Criteria

Inclusion criteria comprised recipes with a clearly defined regional origin (North, Centre, or South), presence in at least one of the selected reference sources, and preservation of original ingredients and traditional cooking methods representative of local gastronomy. An additional inclusion criterion was the availability of complete information on ingredients and respective quantities, which was essential for nutritional analysis. Recipes lacking precise regional identification, those that had undergone substantial modern adaptations (e.g., substitution of traditional ingredients with contemporary alternatives), or those with insufficient quantitative data were excluded.

#### 2.2.3. Nutritional Composition Assessment

For each recipe, nutritional composition per 100 g was determined for the following parameters: energy value (kcal), protein (g), total carbohydrates (g), sugars (g), dietary fibre (g), total fat (g), saturated fatty acids (g), and sodium (mg). These variables allow the assessment of energy density and overall nutritional quality and enable comparison with current European reference values [14]. The number of recipes per region and per dish category was defined to ensure balanced representation of traditional culinary practices while maintaining feasibility within a pilot study framework. This strategy prioritised cultural relevance and methodological transparency over statistical power.

### 2.3. Data Organization

Data collection began with recipe selection, followed by ingredient quantification and calculation of nutritional composition. As many recipes reported household measures rather than standardised units, all ingredient quantities were converted into grams using the reference manual *Pesos e Porções de Alimentos* [15], ensuring consistency and accuracy. Nutritional composition was determined using an online nutritional analysis software, which primarily relies on the Portuguese Food Composition Table [16]. When specific ingredients were not available in this database, nutritional information was obtained from the FatSecret platform (2024) [17], using values derived primarily from manufacturers’ nutrition labels and other publicly available food composition data. A nutritional assessment sheet was created for each recipe from the three regions. All nutritional data were subsequently organised into a table using Microsoft Excel^®^ format for further statistical analysis.

### 2.4. Data Analysis

A database was constructed to operationalise the variables under study, including variable coding, description, scoring, and statistical categorisation. Statistical analysis was performed using SPSS^®^ software, version 29.0 (SPSS Inc., Chicago, IL, USA). Results were expressed as mean ± standard deviation (SD). Data normality was assessed using the Shapiro–Wilk test, which indicated that several variables were not normally distributed in at least one region. Therefore, data were logarithmically transformed (log_10_) to approximate normality. After transformation, the assumptions required for one-way analysis of variance (ANOVA) were reassessed, including normality of distributions and homogeneity of variances (Levene’s test), both of which were confirmed. Comparisons between regions were performed using one-way ANOVA, followed by Tukey’s post hoc test for multiple comparisons. Statistical significance was set at *p* < 0.05. Additionally, six complete menus were constructed (two per region), representing the combinations with the lowest and highest energy values based on the analysed recipes. These menus were evaluated and compared with reference values for energy and nutrient intake. A daily energy intake of 2000 kcal was considered, in accordance with Regulation (EU) No. 1169/2011 [18], with lunch defined as contributing 30% of total daily energy intake [19,20]. Reference macronutrient distribution was set at 15% protein, 55% carbohydrates, and 30% lipids, based on World Health Organization recommendations [21]. For dietary fibre, the EFSA reference value of 25 g/day was adopted [14]. The upper limit for free sugars was defined according to World Health Organization guidelines [22], which recommend that free sugars should not exceed 10% of total daily energy intake. Saturated fatty acids (SFA) were assessed in accordance with EFSA guidance [14], which recommends that their contribution should not exceed 10% of total daily energy intake. Based on these criteria, the following reference values were defined for lunch: 600 kcal energy, 22.5 g protein, 82.5 g carbohydrates, 20 g lipids, 60 g sugars, 7.5 g dietary fibre, 6.7 g SFA, and 600 mg sodium. Results were expressed as percentages of adequacy relative to these reference values, allowing assessment of the degree of compliance of regional menus with current nutritional recommendations. This approach enabled an integrated evaluation of the nutritional quality of traditional Portuguese recipes, both in absolute terms (nutritional composition per 100 g) and relative terms (adequacy in relation to international guidelines).

## 3. Results and Discussion

The results revealed significant nutritional differences among traditional Portuguese first courses from the North, Centre, and South regions (Table 2), reflecting regional variability in ingredients and cooking methods. Comparative analysis identified significant variations in energy value, macronutrient distribution, dietary fibre, sugars, and sodium content, highlighting distinct regional patterns.

Regarding energy value per 100 g, first courses from the Northern region presented the highest mean values (≈202 ± 21 kcal), followed by those from the Southern (≈133 ± 18 kcal) and Central regions (≈126 ± 26 kcal). This difference is primarily attributable to the higher energy density of Northern preparations, which tend to have lower water content and higher proportions of starchy foods, protein, and fat. Representative examples include *Caldo Verde* (potato and chorizo soup), *Bolinhos de Bacalhau* (codfish cakes that absorb oil during frying), *Bola de Carne* (a bread-based preparation with cured meats), and *Rancho* (a dish combining pasta, veal, and cured pork products).

In the Centre, lower energy density is associated with the predominance of soups and vegetable-based dishes, such as *Sopa de Pedra* and *Sopa de Entulho*, despite the inclusion of some fried items, including *Peixinhos da Horta*. In the South, intermediate energy values reflect the frequent consumption of fish- and shellfish-based soups, such as *Sopa de Cação* and *Sopa de Lingueirão*, as well as first courses prepared with moderate amounts of olive oil, such as *Conquilhas à Algarvia*.

With respect to protein content, the highest mean values were observed in the Southern region (≈12 ± 1.9 g), followed by the North (≈11 ± 1.6 g) and the Centre (≈6 ± 1.4 g). The predominance of fish, shellfish, and eggs (foods of high biological value) in Southern first courses explains these findings, as illustrated by preparations such as *Espargos Bravos com Ovos*. In the North, protein intake is mainly derived from meats and cured products, such as *Folar Transmontano* and *Bola de Carne*, as well as from fish-based preparations like *Bolinhos de Bacalhau*. In the Centre, lower protein density is associated with a higher proportion of broths, vegetables, and legumes, which dilute protein content per 100 g, although some dishes still include meat, fish, or cured meats.

Carbohydrate content was highest in the North, followed by the Centre and the South, reflecting the frequent use of potatoes, bread, and flour in Northern recipes. In the Centre, intermediate values are associated with the inclusion of legumes and cereals in soups and salads, whereas in the South, lower carbohydrate content reflects a greater emphasis on fish and vegetables and a reduced contribution from starchy ingredients. Sugar content was low and similar across regions (≈1–2 g), corresponding mainly to naturally occurring sugars in vegetables, such as onions.

Differences in dietary fibre content were modest, although the Centre showed slightly higher values, associated with the frequent use of legumes and vegetables in dishes such as *Sopa de Pedra*, *Sopa de Entulho*, and *Salada de Feijão-Frade*. Regarding total fat, the highest mean values were observed in the Centre (≈14 ± 1.5 g), largely attributable to the predominance of fried preparations such as *Peixinhos da Horta* and *Pataniscas de Bacalhau*, followed by the North (≈11 ± 1.4 g) and the South (≈6 ± 2.6 g). In the North, fat content is mainly derived from meats and cured products (e.g., *chorizo, prosciutto, ham and other cured meats*), whereas in the South, olive oil constitutes the primary lipid source and is generally used in moderate amounts, often combined with lean fish and shellfish.

The profile of SFA followed a similar trend, with higher values in the North and Centre due to the greater use of animal-based ingredients rich in saturated fat. Sodium content was markedly higher in the North (≈482 ± 91.8 mg), reflecting the frequent use of salted and cured meats in first-course preparations, such as *Caldo Verde*, *Folar Transmontano*, *Bola de Carne*, *Rancho*, and *Sopa Seca à Moda do Minho*. In contrast, sodium levels in the Centre (≈ 37 ± 57 mg) and the South (≈136 ± 37 mg) were substantially lower, consistent with the reduced use of processed meats and the predominance of vegetable and fish-based preparations.

In summary, the Northern region was characterised by higher energy density, higher carbohydrate content, and elevated sodium levels, reflecting the frequent use of starchy ingredients, meats, and cured products in less diluted preparations. The Central region exhibited higher total fat values, largely resulting from the prevalence of fried dishes, whereas the Southern region showed a more balanced nutritional profile, with higher protein content and a predominance of unsaturated fats derived from olive oil and fish. Mean SFA content was higher in the North region compared to the Centre and South regions. Sugar content remained low and homogeneous across regions, while dietary fibre showed only minor variation, with a slight advantage observed in the Centre.

The analysis of the average nutritional composition of main courses from the North, Centre, and South regions (Table 3) revealed regional differences associated with ingredient selection, relative proportions of meat, fish, and vegetables, and the cooking techniques characteristic of each area of the country.

Main courses from the Northern region presented the highest mean energy value (≈207 ± 22.8 kcal), followed by those from the Southern (≈179 ± 63.9 kcal) and Central regions (≈173 ± 22.3 kcal). This pattern reflects the predominant types of preparations characteristic of each region. In the North, dishes such as *Rojões à Moda do Minho*, *Arroz de Pato à Moda de Braga*, and *Papas de Sarrabulho à Moda de Sandim do Douro* stand out due to the use of fatty meats and high proportions of starchy ingredients. Variations of *Bacalhau à Gomes de Sá* and *Bacalhau à Zé do Pipo* also exhibit high energy density because of the use of olive oil, mayonnaise, and potatoes.

In the Centre, lower energy values are associated with more balanced and diversified dishes that combine nutrient-dense ingredients with components rich in water and vegetables, thereby reducing energy content per 100 g. Preparations such as *Cozido à Portuguesa* and *Feijoada à Portuguesa* include meats, cured products, and legumes, but also large amounts of vegetables, potatoes, and broth. Similarly, *Caldeirada Rica* and *Lampreia Caseira* incorporate lean fish, tomato, and onion, resulting in more voluminous dishes with lower fat concentration.

In the South, intermediate energy values reflect a balance between richer meat-based dishes, such as *Entrecosto Frito com Migas* and *Pezinhos de Porco de Coentrada*, and lighter fish- and seafood-based recipes, including *Arroz de Berbigão* and *Carapaus Alimados*. Protein content was highest in the Southern region (≈13 ± 7.0 g), followed by the North (≈12 ± 2.0 g) and the Centre (≈11 ± 1.0 g). In the South, fish- and seafood-based dishes predominate, such as *Bifes de Atum em Cebolada*, *Arroz de Berbigão*, and *Lulas Cheias*, providing high biological value protein with relatively low saturated fat content. In the North, protein levels are also elevated due to the frequent use of meat and codfish in dishes such as *Arroz de Pato à Moda de Braga*, *Bacalhau à Gomes de Sá*, and *Bacalhau à Zé do Pipo*. In the Centre, lower protein density per 100 g results from the combination of meat or fish with larger quantities of vegetables, rice, or potatoes.

Carbohydrate content was broadly similar across regions, with a slight upward trend in the North and Centre. In the Centre, dishes such as *Bacalhau à Brás*, *Bacalhau com Broa*, and *Arroz de Lampreia* contribute substantial amounts of starchy foods. In the North, carbohydrate levels are explained by the frequent use of rice, potatoes, and pasta, whereas in the South, slightly lower values were observed, although dishes such as *Arroz de Berbigão*, *Migas à Alentejana*, and *Carapaus Alimados* also contribute to total carbohydrate intake. Sugar content was low and similar across regions, as expected for savoury main courses.

Values for dietary fibre were very similar among regions (≈1 g), with no relevant differences. This uniformity reflects the widespread inclusion of fibre-rich plant-based foods, such as legumes, vegetables, and cereals, across all regions.

Regarding total fat, the highest mean values were observed in the North (≈14 ± 2.2 g), followed by the South (≈11 ± 3.9 g) and the Centre (≈10 ± 2.1 g). In the North, higher fat content results from the use of pork, cured meats, sausages, and lard, as well as the frequent addition of olive oil, as seen in *Papas de Sarrabulho*, *Rojões à Moda do Minho*, and *Arroz de Pato à Moda de Braga*. In the South, fats are predominantly of plant origin, mainly olive oil, which is widely used in regional cuisine. In the Centre, although fat is added, the predominance of broth-based dishes contributes to a lower average lipid density.

The SFA profile was similar across regions, reflecting the combined use of olive oil and fish as major fat sources. Sodium content was relatively homogeneous, ranging from approximately 240 to 270 mg, with slightly higher mean values observed in the Centre (≈272 ± 145 mg), followed by the South (≈269 ± 116 mg) and the North (≈243 ± 126 mg). In the Centre, higher sodium values are associated with the use of sausages, salted meats, and codfish. In the North, although salted ingredients are common, sodium concentration may be partially diluted in preparations with higher oil or milk content. In the South, intermediate sodium values are related to the use of molluscs and shellfish naturally rich in sodium, such as in *Arroz de Berbigão* and *Carne de Porco com Amêijoas*, as well as moderate use of condiments.

Overall, the Northern region stood out for higher energy density and lipid content, reflecting the frequent use of fatty meats, cured products, and culinary fats. The Southern region presented a more favourable lipid profile due to the predominance of unsaturated fats from olive oil and fish. The Central region showed lower energy and lipid density but slightly higher sodium levels, resulting from the combined use of salted meats, sausages, and codfish. Differences in SFA, protein, carbohydrates, and dietary fibre were minimal, suggesting that, regardless of region, traditional Portuguese main courses maintain a relatively balanced nutritional profile for these parameters.

The analysis of the average nutritional composition of traditional Portuguese desserts from the North, Centre, and South regions (Table 4) revealed relatively small differences in energy density and macronutrient distribution, largely associated with the choice and proportion of key ingredients, namely sugar, eggs, flour, nuts, and fats. Desserts from the Northern region showed the highest mean energy value (≈385 ± 28.1 kcal), followed by those from the South (≈375 ± 30.4 kcal) and the Centre (≈374 ± 80.7 kcal). This pattern reflects the predominant use of sugar, almonds, egg yolks, and added fats in dense preparations with low water content. In the North, desserts such as Toucinho-do-Céu (500 g sugar, 250 g almonds, and 50 g butter) and Jesuítas (puff pastry with butter and an egg white–sugar filling) contribute substantially to energy density. Other examples, including *Pão de Ló* and *Pastéis de Santa Clara*, which are rich in sugar and egg yolks, further reinforce this trend.

In the Central region, although desserts such as *Tigeladas* and *Ovos Moles de Aveiro* are also energy-dense, the inclusion of lighter preparations, such as *Carólos*, contributes to a lower regional average. In the Southern region, intermediate energy values result from a balance between highly energetic desserts, such as *Morgado de Figo* (500 g figs, 500 g sugar, and 500 g almonds), and lighter options, such as *Pudim de Água*, in which the addition of water slightly reduces overall energy density. Protein content was highest in the South, followed by the North and the Centre, reflecting differences in the number of eggs used in regional dessert recipes. Carbohydrates were the predominant macronutrient in desserts from all regions, although with marked regional variation.

The highest carbohydrate content was observed in the South, followed by the North, and the lowest in the Centre. In the South, this pattern is attributable to the extensive use of refined sugar and naturally sweet ingredients, as seen in *Morgado de Figo*, *Bolinhos de D. Rodrigo* (1 kg sugar), and *Tarte de Claras*. In the North, carbohydrate levels are similarly high due to desserts such as *Toucinho-do-Céu*, *Pastéis de Santa Clara*, and *Pão de Ló*, which incorporate large amounts of sucrose and flour. In contrast, the lower carbohydrate content observed in the Centre reflects a more diversified ingredient composition, where sugar—although abundant in desserts such as *Arroz Doce Saloio* and *Ovos Moles de Aveiro*—is combined with larger proportions of milk, flour, and eggs, resulting in lower carbohydrate concentration per 100 g.

Values for dietary fibre were low across all regions, ranging from 0 to 2 g. The North and South presented values closer to 2 g, mainly due to the inclusion of flour, figs, and nuts. In the Centre, fibre content was nearly negligible, reflecting the predominance of sugar, milk, and eggs in desserts such as *Farófias*, *Ovos Moles de Aveiro*, and *Arroz Doce Saloio*. Regarding total fat, the highest mean values were observed in the Centre (≈23 ± 10.0 g), followed by the North (≈18 ± 3.0 g) and the South (≈13 ± 2.9 g). In the Centre, this pattern is largely driven by desserts rich in added fats, such as *Areias*, which contain lard; the low water content and high sugar concentration of these preparations limit fat dilution in the final product. In the North, intermediate fat values result from the use of butter and lard in puff pastry and traditional cakes, such as *Jesuítas* and *Rosquilhas*. In the South, lower total fat content reflects the use of smaller amounts of added fats, with a greater contribution from natural lipid sources, such as olive oil and almonds. Although these ingredients contribute to overall energy value, they provide a more favourable lipid profile. The SFA content followed the sequence North (≈6 g) > Centre (≈4 g) > South (≈2 g), reflecting the greater use of animal fats in Northern and Central desserts and the predominance of unsaturated fats from olive oil and almonds in Southern preparations. Sodium content was very low across all regions, with slightly lower values observed in the South (≈37 mg), followed by the Centre (≈41 mg) and the North (≈73 mg). As expected, salt is not a relevant ingredient in desserts, and the minor variations observed are attributable to raw materials such as eggs and butter or to differences in preparation processes.

Overall, the Northern region stood out for higher energy density and saturated fat content, with intermediate total lipid values. The Central region exhibited the highest total fat content, intermediate SFA levels, and the lowest dietary fibre content. The Southern region showed higher protein, carbohydrate, and sugar contents but lower total fat and SFA levels, while maintaining residual sodium values. These results reflect regional pastry traditions and ingredient matrices, particularly the relative proportions of eggs, sugar, added fats, flour, and nuts that characterise traditional Portuguese desserts.

To determine whether the descriptive differences observed between regions were statistically significant, an inferential comparison of the mean nutritional values of the three regions was performed (Table 5). This analysis aimed to identify which nutritional parameters of traditional Portuguese recipes differed significantly between the North, Centre, and South regions. Statistically significant regional differences were observed only for first-course dishes. For energy value, a significant difference was identified between the North (202 ± 21 kcal) and the Centre (126 ± 26 kcal) (*p* < 0.05), whereas values for the South (133 ± 18 kcal) did not differ significantly from either region.

Regarding dietary fibre, a significant difference was observed between the Centre (2.0 ± 0.5 g) and the South (1.0 ± 0.3 g), with the Centre showing the highest mean value. With respect to sodium, the North (482 ± 91.8 mg) differed significantly from both the Centre (137 ± 57.2 mg) and the South (136 ± 37.3 mg), presenting markedly higher sodium content.

No statistically significant regional differences were observed for protein, carbohydrates, sugars, total fat, or SFA in first-course dishes (*p* > 0.05). Furthermore, for main courses and desserts, no statistically significant differences were identified for any of the evaluated nutritional parameters (*p* > 0.05). Although descriptive variability was observed between regions, these differences were insufficient to reach statistical significance, suggesting a broadly similar nutritional composition of main courses and desserts across the three regions.

A comparison was also conducted between the nutritional values of the most and least energy-dense complete menus from the North, Centre, and South regions, with the aim of assessing their adequacy relative to established nutritional recommendations for a complete meal. This analysis, summarised in Table 6, complements the previous inferential evaluation by providing an applied perspective on the nutritional impact of traditional dishes when combined into representative regional menus.

The menus were constructed based on the recipes previously analysed, integrating a first course, a main course, and a dessert from each region to reflect the typical structure of a complete meal. This approach allowed assessment of the extent to which combinations of traditional dishes align with, or deviate from, reference values for a balanced meal, thereby identifying potential nutritional excesses or deficits and highlighting regional differences in Portuguese dietary patterns.

The complete menus analysed in this study represent theoretical combinations based on standardised nutritional values per 100 g of each recipe. These combinations were not intended to replicate actual portion sizes or real consumption patterns, but rather to provide a comparative framework for evaluating the nutritional balance of traditional dishes when combined into a complete meal.

Nutritional adequacy of the complete menus was assessed using the Comparison with Reference Value (CRV), which expresses each nutritional parameter as a percentage of the reference value established for a complete meal. Values between 90–110% were considered adequate for energy and macronutrients. For sugars, SFA, and sodium, values exceeding the reference were classified as excessive, whereas for dietary fibre, values below 90% of the reference were considered insufficient.

In the Northern region, the least energy-dense menu presented an adequate energy value (97%) but showed marked macronutrient imbalances, including deficits in carbohydrates (61%) and dietary fibre (67%), alongside excesses of total fat (155%) and sodium (124%). This pattern reflects the characteristic regional culinary profile, marked by the frequent use of fatty meats, sausages, and added fats, combined with a lower contribution from cereals and vegetables.

In contrast, the most energy-dense Northern menu exhibited generalised excesses across almost all parameters, namely energy (182%), protein (133%), total fat (360%), SFA (419%), and sodium (149%), together with a pronounced dietary fibre deficit (40%). These findings highlight the high energy and lipid density of traditional Northern meals, largely associated with abundant use of animal fats and limited inclusion of fibre-rich foods.

In the Central region, the least energy-dense menu showed substantial inadequacies, with deficits in energy (43%), protein (84%), carbohydrates (35%), dietary fibre (53%), and total fat (35%), indicating an overall insufficient nutritional profile. The remaining parameterssugars (38%), SFA (15%), and sodium (71%)—remained within acceptable limits. This profile corresponds to a light, low-density meal with reduced energy and macronutrient contribution per 100 g of food. Conversely, the most energy-dense Central menu largely exceeded the energy reference value (188%) and showed inadequate levels of carbohydrates (68%), dietary fibre (40%), and SFA (224%), while protein (90%), sugars (28%), total fat (73%), and sodium (62%) remained within recommended limits. These results indicate that richer Central menus may provide excessive energy and disproportionate saturated fat, while still presenting deficits in carbohydrates and dietary fibre.

Menus from the Southern region displayed the most contrasting nutritional profiles. The least energy-dense menu, corresponding to 74% of the energy reference value, revealed very high protein content (236%) alongside deficits in carbohydrates (50%), sugars (42%), dietary fibre (13%), total fat (75%), and SFA (15%), while sodium remained within adequate limits (22%). In contrast, the most energy-dense Southern menu exceeded energy recommendations (171%) and showed very high levels of protein (324%), total fat (275%), and SFA (164%), accompanied by deficits in carbohydrates (63%), sugars (58%), and dietary fibre (53%), and slightly elevated sodium values (111%). This pattern characterises a meal with high protein and lipid density, reflecting the combined use of meat, fish, and eggs and a relatively lower proportion of plant-based foods.

Overall, the most energy-dense menus exceeded recommended energy values, demonstrating the high energy density of traditional Portuguese meals regardless of region. Northern menus were particularly unbalanced, with excessive energy, fat, SFA, and sodium and insufficient dietary fibre, reflecting the frequent use of fatty meats and animal fats. In the Central region, wide variation was observed between menus, with the least energy-dense menu showing generalised deficits in energy and macronutrients, and the most energy-dense menu exceeding energy and SFA recommendations while remaining deficient in carbohydrates and fibre. In the Southern region, a markedly hyperproteic profile was observed, especially in the most energy-dense menus, which may reflect the simultaneous consumption of meat, fish, and eggs commonly found in regional gastronomy.

Comparison with reference values indicates that, despite the cultural and gastronomic importance of traditional Portuguese recipes, complete traditional menus frequently exceed recommended energy limits and present nutritional imbalances. These findings highlight the importance of promoting nutritionally balanced adaptations of traditional dishes, particularly through reductions in fat and salt content and increased inclusion of vegetables, legumes, and whole grains, to preserve gastronomic identity while aligning with current dietary recommendations. The Reference Meal Values were used exclusively as standardized comparative benchmarks to allow objective comparison among complete menus and were not intended to represent individual nutritional requirements, which naturally vary according to age, sex, physical activity, and physiological condition. Consequently, the present findings should be interpreted as comparative estimates of the nutritional profiles of representative traditional recipes rather than precise estimates of individual dietary intake.

Unlike previous publications that primarily describe Portuguese gastronomy from historical, cultural, or culinary perspectives, the present study provides a standardized nutritional comparison of representative traditional recipes and complete regional menus using a common analytical framework. This approach enables direct comparison between Portuguese regions under the same methodological criteria and identifies region-specific nutritional patterns that may support future recipe reformulation, nutritional surveillance, and the expansion of Portuguese food composition data for traditional composite dishes.

Despite these nutritional limitations, traditional Portuguese recipes represent a cultural and gastronomic heritage of high symbolic value, closely linked to territory, local resources, and eating habits transmitted across generations. Therefore, preserving recipe authenticity while introducing gradual and culturally sensitive nutritional adjustments is essential to reduce excess fat, salt, and sugar without compromising characteristic flavours or cultural identity.

When situated within the international literature on traditional dietary patterns, the findings of the present study support that traditional Portuguese cuisine is historically linked to the Mediterranean dietary pattern [23,24], while also highlighting important deviations with nutritional relevance. Core elements of the Mediterranean diet, such as the use of olive oil as the primary culinary fat, frequent consumption of fish and seafood, and the inclusion of vegetables and legumes, were clearly reflected in the Portuguese dishes analyzed, particularly in the Southern region, which exhibited a more beneficial lipid profile and higher-quality protein sources [24,25]. However, the present results demonstrate that, despite this Mediterranean culinary basis, traditional Portuguese meals often diverge from the Mediterranean dietary model as defined in contemporary dietary guidelines, notably through the frequent combination of multiple energy-dense dishes within a single meal and the widespread use of cured and salted meats in certain regions. It is therefore important that contemporary traditional Portuguese cuisine incorporates preparation practices and menu choices that enhance its potential health benefits. This highlights the relevance of region-specific reformulation strategies that reinforce Mediterranean principles already inherent in Portuguese culinary tradition, rather than introducing external dietary models.

Given the observed imbalances in the nutritional profiles of the analyzed traditional dishes, several studies [26,27,28] have highlighted strategies to improve diet quality by making use of local raw materials, traditional food biodiversity, and culinary techniques. Research on nutrient profiling of traditional dishes suggests that transforming traditional food patterns towards more plant-based and diverse ingredient use can support healthier eating styles while maintaining cultural heritage, for example through adapting traditional recipes to include a greater variety of plant-based ingredients while preserving local food culture, thereby increasing the intake of fruits, vegetables, and seasonal plant consumption [26]. Experimental work comparing traditional and less aggressive cooking methods has also demonstrated that modifying culinary techniques can reduce nutrient losses and improve the overall nutritional quality of prepared foods [29]. Community-based studies point out the importance of promoting traditional foods within local agroecological systems to enhance both nutritional health and food security by increasing the diversity and frequency of nutrient-rich local foods in diets [26]. Moreover, integrating modern nutritional recommendations with culturally traditional ingredients, such as incorporating plant-based proteins or micronutrient-rich local crops, can help reconcile nutritional adequacy with cultural dietary identity [30]. These approaches provide a substantive basis for future recommendations on how traditional Portuguese recipes might be modified or complemented with local ingredients and techniques to achieve improved nutritional profiles in culturally respectful ways.

In practical terms, several culturally coherent adaptations could contribute to improving the nutritional balance of traditional Portuguese dishes while preserving their regional identity. In Northern recipes characterized by higher sodium and lipid content, the quantity of cured meats (e.g., chorizo or ham) could be gradually reduced or partially replaced by fresh meat cuts or plant-based ingredients such as beans or chickpeas, which are already integral to Portuguese culinary traditions. Increasing the proportion of vegetables typical of the region, including cabbage, turnip greens, onions, and other seasonal products, in soups such as Caldo Verde could also improve dietary fiber density while reducing overall energy concentration. These strategies are consistent with evidence suggesting that increasing plant-based components and food diversity in traditional diets contributes to improved nutritional quality without compromising cultural authenticity [26,30,31]. Moreover, historical dietary patterns often reflect the adaptation of local cuisines to environmental conditions and nutritional needs, such as the consumption of nutrient-dense foods rich in fat-soluble vitamins, minerals, and energy in colder regions or in populations engaged in physically demanding activities [7,14,16,17,21,22,31]. The frequent use of pork products, leafy vegetables, and legumes in Northern Portuguese cuisine may therefore partly reflect traditional strategies to ensure adequate intake of micronutrients such as iron, zinc, and fat-soluble vitamins within locally available food systems.

In the Central region, where fried preparations contribute substantially to total fat content, traditional recipes such as Peixinhos da Horta or Pataniscas de Bacalhau could be adapted using alternative culinary techniques, including oven baking or air frying, which reduce oil absorption while preserving sensory characteristics. Previous studies have demonstrated that modifying cooking techniques and reducing discretionary fat and salt during preparation can significantly improve the nutritional profile of traditional foods while maintaining their acceptability [29,32,33,34]. When changes to raw materials are limited due to culinary tradition or product availability, technological adjustments in food preparation—such as steaming, baking, or controlled-temperature cooking—have been shown to enhance nutrient retention and reduce excessive fat intake while preserving the cultural identity of traditional dishes [29,33].

In Southern cuisine, which already reflects several Mediterranean dietary principles, further improvements could be achieved by increasing the proportion of seasonal vegetables, pulses, and whole cereals (e.g., whole-grain bread in açordas) and by moderating the simultaneous inclusion of multiple animal protein sources within the same meal. Reinforcing these plant-based components aligns with current evidence supporting the health benefits of Mediterranean dietary patterns and their emphasis on vegetables, legumes, whole grains, and olive oil as key elements of a balanced diet [23,24,25,35,36,37,38]. Such adaptations are also consistent with research highlighting that strengthening plant-based diversity within traditional food systems can simultaneously improve diet quality, nutritional adequacy, and sustainability without compromising cultural heritage [26,27,28].

Overall, these strategies rely on locally available raw materials and culinary practices already embedded in Portuguese food culture. Gradual reformulation through modest ingredient substitutions, increased plant-based diversity, and improved cooking techniques has been widely recognized as an effective approach to enhance the nutritional quality of traditional dishes while preserving their sensory characteristics and cultural significance [27,28,31,36]. Thus, these adaptations not only preserve cultural identity but also align with the population’s specific nutritional needs, including liposoluble vitamins, key minerals, and other nutrients, and can be further enhanced by modern preparation techniques that maximize nutrient retention [14,16,17,21,22].

Regional epidemiological evidence indicates that the burden of major chronic diseases and their risk factors is not evenly distributed across Portugal, reflecting underlying differences in lifestyle, dietary habits, and socioeconomic conditions. Data from the First Portuguese National Health Examination Survey (INSEF 2015) show high prevalences of hypertension (≈36%), overweight (≈39%), and obesity (≈29%) among adults aged 25–74 years, with regional variations associated with sociodemographic factors such as age, education, and urbanization [39]. Specifically, the Northern region exhibited a higher age-standardized prevalence of diabetes (≈9.8%) compared with the Centre (≈8.3%) and the Algarve (≈7.7%) [40], while obesity also displayed a geographically uneven distribution, being higher in the North and Centre, with notable gender differences between urban and rural areas [41,42].

The results of the present study provide nutritional context for these epidemiological patterns by suggesting that regional differences in traditional menu composition may contribute to variations in exposure to dietary risk factors associated with chronic disease. Northern menus showed high energy density, excessive total fat, saturated fatty acids (SFA), and sodium, and low dietary fiber, reflecting the frequent consumption of fatty meats and animal products—dietary features that have been associated with higher prevalence of hypertension, obesity, insulin resistance, and type 2 diabetes in population cohorts and nutritional epidemiology studies [43,44,45]. In the Centre, wide variability was observed among menus, with less energy-dense menus deficient in energy and macronutrients and more energy-dense menus exceeding recommended energy and SFA intakes, yet still insufficient in carbohydrates and fibre, suggesting nutritional imbalances that align with evidence linking suboptimal macronutrient distributions to adverse cardiometabolic outcomes [46,47,48]. In the South, high-protein profiles were observed in both the less and more energy-dense menus, with the highest protein content recorded in the most energy-dense menu. This markedly hyperproteic profile may reflect the simultaneous consumption of meat, fish, and eggs commonly found in regional gastronomy. Although the most energy-dense Southern menu exceeded the Reference Meal Value (RMV) for saturated fatty acids, its saturated fat content was substantially lower than that of the most energy-dense Northern menu. Overall, these findings indicate that the Southern menus exhibited a distinct nutritional profile characterized by high protein content across both menu types and comparatively lower saturated fat content than the corresponding Northern menu. These observations are consistent with previous studies reporting that dietary patterns characterized by higher protein intake and lower saturated fat are generally associated with more favourable cardiovascular and metabolic risk profiles [49,50,51]. Overall, the findings indicate that, although traditional Portuguese menus retain important cultural value, their nutritional composition may expose populations to region-specific dietary risk factors, particularly related to excessive energy, saturated fat, sodium, and insufficient fiber intake. Therefore, the regional dietary patterns identified in this study provide a plausible explanatory framework for some of the epidemiological differences observed in Portugal and highlight the need for region-specific public health interventions. Such strategies could include the promotion of nutritionally balanced adaptations of traditional recipes, with gradual reductions in fat and salt and increased inclusion of vegetables, legumes, and whole grains, while simultaneously preserving gastronomic identity and reinforcing the principles of the Mediterranean dietary pattern associated with reduced cardiometabolic risk [24,25,52,53,54].

Some limitations of this study should be acknowledged. First, although 60 traditional Portuguese recipes were analysed, expanding the sample size would be important to verify the consistency of the observed patterns. Additionally, the primary bibliographic reference used for recipe selection frequently lacked detailed ingredient quantity information, preventing nutritional analysis of certain preparations. When the recipe taken from Maria de Lourdes Modesto’s Traditional Portuguese Cooking book (1981) [4] did not have all the quantities of ingredients clearly described, the traditional recipe for the same typical dish described in the magazine Teleculinária (2024) [11] was used. It should also be noted that, although the Portuguese Food Composition Table served as the primary reference source, complementary databases were consulted when necessary. This approach may introduce minor variability in nutrient estimation; however, it reflects common practice in the nutritional analysis of composite dishes and was applied consistently across all regions.

Nutritional composition was estimated using standardized food composition databases, primarily the Portuguese Food Composition Table, whose nutrient values represent mean values derived predominantly from laboratory analyses and are intended for nutritional assessment and research purposes. Whenever available, nutrient values for foods as consumed (e.g., cooked foods) were obtained directly from the database. Therefore, the reported values partially reflect the natural variability observed across food samples. Nevertheless, they should be interpreted as standardized estimates rather than exact values for every individual recipe preparation, since additional variability related to ingredient selection, geographical origin, seasonality, and cooking practices cannot be fully accounted for. Nevertheless, because the same standardized methodology and nutrient databases were applied consistently to all recipes, these limitations are unlikely to have affected the validity of the comparative analyses between regions, which constituted the primary objective of the study.

This study provides a foundation for the development of nutritionally improved versions of traditional Portuguese recipes, based on balanced reformulation strategies such as reducing fat and salt, increasing the proportion of vegetables, legumes, and whole grains, and adopting cooking methods requiring less added fat. Future research should aim to increase the number of analysed recipes per region, include the autonomous regions of the Azores and Madeira, and thus achieve a more comprehensive characterisation of national gastronomy. Additionally, population-based surveys assessing public perception of the nutritional quality and energy content of traditional dishes across different age groups could contribute to the design of culturally adapted nutrition education strategies. This approach would allow a better understanding of how traditional gastronomy is perceived and valued by the population, supporting the development of targeted interventions that promote healthier eating habits while respecting cultural identity. Such strategies may help bridge the gap between traditional culinary practices and contemporary nutritional recommendations, fostering sustainable dietary patterns that preserve Portuguese gastronomic heritage while addressing current public health challenges.

## 4. Conclusions

The present study is the first to compare the nutritional composition of 60 traditional Portuguese recipes from three different regions, providing an in-depth and comparative analysis of Portuguese gastronomy from the North, Centre, and South. The findings identify region-specific nutritional patterns using a standardized analytical framework, providing one of the first systematic nutritional comparisons of representative traditional Portuguese recipes across the three mainland regions.

Despite a shared culinary heritage, traditional Portuguese dishes exhibited marked regional variability with important public health implications. Northern menus were characterized by the highest sodium content, with the least energy-dense complete menu providing 741 mg of sodium (124% of the Reference Meal Value, RMV), compared with only 129 mg (22% of the RMV) in the corresponding Southern menu. This pattern is consistent with the frequent use of cured meats, sausages, and salted products in traditional Northern preparations, such as Caldo Verde, Rancho, and Bola de Carne, highlighting these dishes as priorities for nutritional reformulation. This finding is particularly relevant in light of international recommendations aimed at reducing sodium intake to prevent hypertension and cardiovascular disease. In addition, the most energy-dense complete menus ranged from 923 kcal in the South to 1130 kcal in the Centre, all substantially exceeding the RMV of 600 kcal, while dietary fiber remained below the RMV in all complete menus, ranging from 1 to 5 g per meal, well below the recommended 7.5 g. These comparisons were based on standardized Reference Meal Values used exclusively as comparative benchmarks and should not be interpreted as individualized dietary recommendations or estimates of actual food intake.

Taken together, these findings demonstrate that nutritional imbalances are neither homogeneous across regions nor consistent across dish categories, reinforcing the need for region-specific and dish-specific approaches rather than generic dietary recommendations. Consequently, the reformulation of traditional Portuguese gastronomy should be grounded in robust scientific evidence and culturally sensitive strategies that address the specific nutritional priorities identified in each region. In the Northern region, priority should be given to gradually reducing the use of cured meats and other high-sodium ingredients, replacing them where possible with lower-sodium alternatives, enhancing flavour through herbs, vegetables, and spices, and increasing the proportion of vegetables and broth while preserving the characteristic sensory profile and cultural authenticity of these preparations. Similar evidence-based adaptations should be tailored to the nutritional priorities identified in the remaining regions, enabling gradual improvements in nutritional quality while preserving the authenticity and sustainability of traditional Portuguese cuisine. These recommendations derive directly from the nutritional imbalances identified in the present analyses and therefore provide evidence-based priorities for future reformulation strategies.

Overall, although Portuguese gastronomy represents an important cultural heritage, many traditional complete meals were characterized by excessive energy density, elevated total fat, saturated fatty acids, and sodium, together with insufficient dietary fiber. When consumed as complete menus without consideration of their overall nutritional balance, these characteristics may contribute to dietary inadequacies and increase the risk of diet-related chronic diseases. Despite its exploratory design, the study provides the first standardized comparative assessment of representative traditional Portuguese recipes across the three mainland regions, generating evidence that may support nutrition policy, recipe reformulation, food composition databases, and future research.

Enhancing the nutritional quality of traditional Portuguese dishes while safeguarding their cultural heritage is essential to promote healthier, more balanced, and sustainable dietary patterns in contemporary Portuguese society.

## Figures and Tables

**Table 1:** Identification of Portuguese culinary recipes used for nutritional composition analysis in three regions of Portugal.

Region	Recipe Code	Recipe Name
North	First Course 1	Caldo-Verde
North	First Course 2	Folar Transmontano
North	First Course 3	Bolinhos de bacalhau
North	First Course 4	Bola de carne
North	First Course 5	Rancho
North	First Course 6	Sopa Seca à Moda do Minho
North	Main Course 1	Rojões à moda do Minho
North	Main Course 2	Arroz de pato à moda de Braga
North	Main Course 3	Lombo de porco assado
North	Main Couse 4	Papas de sarrabulho à moda de Sandim do Douro
North	Main Course 5	Bacalhau à Gomes de Sá
North	Main Course 6	Bacalhau à Zé do pipo
North	Main Course 7	Arroz de Lampreia
North	Main Course 8	Polvo assado na brasa
North	Dessert 1	Sarrabulho doce
North	Dessert 2	Rosquilhas
North	Dessert 3	Pastéis de Santa Clara
North	Dessert 4	Pão de ló
North	Dessert 5	Toucinho-do-céu
North	Dessert 6	Jesuítas
Center	First Course 1	Sopa de entulho
Center	First Course 2	Salada de feijão frade
Center	First Course 3	Peixinhos da horta
Center	First Course 4	Pipis
Center	First Course 5	Sopa de Pedra
Center	First Course 6	Pataniscas de bacalhau
Center	Main Course 1	Feijoada à Portuguesa
Center	Main Course 2	Cabrito assado
Center	Main Course 3	Bife à café
Center	Main Course 4	Cozido à portuguesa
Center	Main Course 5	Bacalhau à Brás
Center	Main Course 6	Bacalhau assado com broa
Center	Main Course 7	Lampreia Caseira
Center	Main Course 8	Caldeirada rica
Center	Dessert 1	Arroz doce saloio
Center	Dessert 2	Farófias
Center	Dessert 3	Areias
Center	Dessert 4	Tigeladas
Center	Dessert 5	Ovos-moles de Aveiro
Center	Dessert 6	Carólos
South	First Course 1	Gaspacho
South	First Course 2	Sopa de cação
South	First Course 3	Sopa de Lingueirão
South	First Course 4	Choquinhos fritos com tinta
South	First Course 5	Conquilhas à Algarvia
South	First Course 6	Espargos bravos com ovos
South	Main Course 1	Carne de porco com amêijoas
South	Main Course 2	Ovos escalfados com ervilhas
South	Main Course 3	Pezinhos de Porco de Coentrada
South	Main Course 4	Entrecosto frito com migas à Alentejana
South	Main Course 5	Bifes de atum em cebolada
South	Main Course 6	Carapaus Alimados
South	Main Course 7	Lulas cheias
South	Main Course 8	Arroz de berbigão
South	Dessert 1	Pudim de água
South	Dessert 2	Morgado de figo
South	Dessert 3	Tarte de claras ou colchão de noiva
South	Dessert 4	Bolo mimoso
South	Dessert 5	Bolos de D. Rodrigo
South	Dessert 6	Queijinhos de amêndoa

**Table 2:** Nutritional composition (per 100 g) of traditional Portuguese first courses from the North, Centre, and South regions.

Region	Recipe	E (kcal)	P (g)	C (g)	S (g)	DF (g)	L (g)	SFA (g)	Na (mg)
North	FC1	165	5	12	1	2	10	3	312
	FC2	305	11	27	1	1	17	6	457
	FC3	178	9	4	1	0	13	2	329
	FC4	193	16	14	1	1	8	2	518
	FC5	186	10	16	1	2	9	2	365
	FC6	184	14	10	1	2	9	3	912
	M ± SD	202 ± 21.0	11 ± 1.6	14 ± 3.1	1 ± 0.0	1 ± 0.3	11 ± 1.4	3 ± 0.6	482 ± 91.8
Centre	FC1	41	2	4	1	3	1	0	152
	FC2	103	5	9	1	3	4	1	124
	FC3	120	3	12	2	2	6	1	15
	FC4	100	11	1	1	0	5	0	3
	FC5	162	8	9	1	3	10	3	392
	FC6	229	6	26	2	2	11	2	138
	M ± SD	126 ± 26.0	6 ± 1.4	10 ± 1.6	1 ± 0.2	2 ± 0.5	14 ± 1.5	1 ± 0.5	137 ± 57.2
South	FC1	120	3	20	3	2	2	0	197
	FC2	109	15	7	0	1	2	0	162
	FC3	120	12	9	1	1	3	1	49
	FC4	204	16	0	0	0	16	2	167
	FC5	79	13	2	0	0	2	0	0
	FC6	166	11	1	0	0	13	3	238
	M ± SD	133 ± 18.2	12 ± 1.9	7 ± 3.1	1 ± 0.5	1 ± 0.3	6 ± 2.6	1 ± 0.5	136 ± 37.3

The basis for calculating the standard deviation is sample-based. Abbreviations: E, energy; C, carbohydrates; DF, dietary fiber; FC, first course; L, total lipids; M, mean; Na, sodium; P, protein; SFA, saturated fatty acids; SD, standard deviation; S, sugars.

**Table 3:** Nutritional composition (per 100 g) of traditional Portuguese main courses from the North, Centre, and South regions.

Region	Recipe	E (kcal)	P (g)	C (g)	S (g)	DF (g)	L (g)	SFA (g)	Na (mg)
North	MC1	210	10	12	1	1	12	4	93
	MC2	207	18	16	0	0	8	3	446
	MC3	265	20	1	1	0	19	5	333
	MC4	231	15	1	0	0	18	5	186
	MC5	120	7	6	2	1	8	1	339
	MC6	184	4	7	2	1	15	2	110
	MC7	312	12	10	0	0	24	5	278
	MC8	130	8	8	1	1	7	1	157
	M ± SD	207 ± 22.8	12 ± 2.0	8 ± 1.8	1 ± 0.3	1 ± 0.2	14 ± 2.2	3 ± 0.6	243 ± 125.7
Centre	MC1	218	6	7	1	2	18	6	516
	MC2	135	15	0	0	0	8	2	191
	MC3	125	12	2	1	0	8	3	151
	MC4	155	14	7	1	1	8	2	281
	MC5	234	11	13	0	2	15	6	475
	MC6	181	8	20	2	3	6	1	146
	MC7	263	11	14	0	1	18	4	201
	MC8	74	11	2	1	1	2	0	211
	M ± SD	173 ± 22.3	11 ± 1.0	8 ± 2.5	1 ± 0.3	1 ± 0.4	10 ± 2.1	3 ± 0.8	272 ± 144.7
South	MC1	121	15	2	0	0	6	1	136
	MC2	140	10	5	2	2	9	3	223
	MC3	271	28	3	0	0	16	7	486
	MC4	275	14	16	0	0	17	5	342
	MC5	130	14	1	1	0	8	1	275
	MC6	147	5	12	1	1	8	1	129
	MC7	137	8	2	1	1	11	2	311
	MC8	209	9	20	0	1	10	2	253
	M ± SD	179 ± 63.9	13 ± 7.0	8 ± 7.3	1 ± 0.7	1 ± 0.7	11 ± 3.9	3 ± 2.2	269 ± 115.8

The basis for calculating the standard deviation is sample-based. Abbreviations: E, energy; C, carbohydrates; DF, dietary fiber; L, total lipids; MC, main course; M, mean; Na, sodium; P, protein; SFA, saturated fatty acids; SD, standard deviation; S, sugars.

**Table 4:** Nutritional composition of desserts from Portuguese gastronomy.

Region	Recipe	E (kcal)	P (g)	C (g)	S (g)	DF (g)	L (g)	SFA (g)	Na (mg)
North	D1	295	11	32	25	2	13	2	90
	D2	399	5	39	16	1	18	3	28
	D3	401	8	54	32	2	17	6	71
	D4	313	9	47	33	1	10	2	48
	D5	428	8	50	45	3	21	5	45
	D6	475	7	42	12	2	31	17	156
	M ± SD	385 ± 28.1	9 ± 0.8	44 ± 3.3	27 ± 4.9	2 ± 0.3	18 ± 3.0	6 ± 2.3	73 ± 18.8
Centre	D1	505	3	30	22	0	42	6	92
	D2	638	3	16	15	0	63	9	34
	D3	441	2	85	74	1	10	3	1
	D4	144	6	22	21	0	4	1	61
	D5	368	8	48	48	0	16	4	25
	D6	148	4	29	17	1	2	1	33
	M ± SD	374 ± 80.7	4 ± 0.9	38 ± 10.3	33 ± 9.6	0 ± 0.2	23 ± 10.0	4 ± 1.3	41 ± 12.9
South	D1	389	2	85	85	0	4	1	7
	D2	417	8	50	46	4	19	2	28
	D3	244	25	37	25	1	7	0	113
	D4	335	8	44	35	2	14	2	48
	D5	416	6	70	70	1	13	3	16
	D6	448	29	49	35	4	23	2	10
	M ± SD	375 ± 30.4	13 ± 4.5	56 ± 7.4	49 ± 9.5	2 ± 0.7	13 ± 2.9	2 ± 0.4	37 ± 16.4

The basis for calculating the standard deviation is sample based. Abbreviations: E, energy; P, protein; C, carbohydrates; D, dessert; DF, dietary fibre; L, total lipids; M, mean; Na, sodium; SFA, saturated fatty acids; SD, standard deviation; S, sugars.

**Table 5:** Nutritional mean values comparison of first courses, main courses and desserts from three Portuguese regions.

Nutritional Composition	North	Centre	South	*p* Value
First Courses (n = 6)				
Energy	202 ^a^ ± 21.0	126 ^b^ ± 26.1	133 ^ab^ ± 18.2	0.02
Protein	11 ^a^ ± 1.6	6 ^a^ ± 1.4	12 ^a^ ± 1.9	0.09
Carbohydrate	14 ^a^ ± 3.1	7 ^a^ ± 1.6	7 ^a^ ± 3.1	0.26
Sugars	1 ^a^ ± 0.0	1 ^a^ ± 0.2	1 ^a^ ± 0.5	0.08
Dietary Fiber	1 ^a^ ± 0.3	2 ^b^ ± 0.5	1 ^a^ ± 0.3	0.02
Lipids	11 ^a^ ± 1.4	6 ^a^ ± 1.5	6 ^a^ ± 2.6	0.13
Saturated Fatty Acids	3 ^a^ ± 0.6	1 ^a^ ± 0.5	1 ^a^ ± 0.5	0.50
Sodium	482 ^a^ ± 91.8	137 ^b^ ± 57.2	136 ^b^ ± 37.3	0.03
Main Courses (n = 8)				
Energy	207 ^a^ ± 64.4	173 ^a^ ± 63.0	179 ^a^ ± 64.0	0.54
Protein	12 ^a^ ± 5.6	11 ^a^ ± 2.9	13 ^a^ ± 7.0	0.77
Carbohydrate	8 ^a^ ± 5.2	8 ^a^ ± 7.0	8 ^a^ ± 7.3	0.99
Sugars	1 ^a^ ± 0.8	1 ^a^ ± 0.7	1 ^a^ ± 0.7	0.79
Dietary Fiber	1 ^a^ ± 0.5	1 ^a^ ± 1.0	3 ^a^ ± 5.4	0.40
Lipids	14 ^a^ ± 6.2	10 ^a^ ± 5.9	13 ^a^ ± 7.5	0.55
Saturated Fatty Acids	3 ^a^ ± 1.8	3 ^a^ ± 2.2	3 ^a^ ± 2.2	0.88
Sodium	243 ^a^ ± 126.0	272 ^a^ ± 145.0	269 ^a^ ± 116.0	0.90
Desserts (n = 6)				
Energy	385 ^a^ ± 28.1	374 ^a^ ± 80.7	375 ^a^ ± 30.4	0.76
Protein	9 ^a^ ± 0.8	4 ^a^ ± 0.9	13 ^a^ ± 4.5	0.09
Carbohydrate	44 ^a^ ± 3.3	38 ^a^ ± 10.3	56 ^a^ ± 7.4	0.14
Sugars	27 ^a^ ± 4.9	33 ^a^ ± 9.6	49 ^a^ ± 9.5	0.15
Dietary Fiber	2 ^a^ ± 0.3	0 ^a^ ± 0.2	2 ^a^ ± 0.7	0.34
Lipids	18 ^a^ ± 3.0	23 ^a^ ± 10.0	13 ^a^ ± 2.9	0.70
Saturated Fatty Acids	6 ^a^ ± 2.3	4 ^a^ ± 1.3	2 ^a^ ± 0.4	0.24
Sodium	73 ^a^ ± 18.8	41 ^a^ ± 12.9	37 ^a^ ± 16.4	0.27

Values in the same row marked with different superscript letters (a, b) are statistically different according to Tukey’s post hoc test (*p* < 0.05). The basis for calculating the standard deviation is sample-based.

**Table 6:** Nutritional composition (per 100 g) and adequacy of the least and most energy-dense complete menus (CRV%) from the North, Centre, and South regions of Portugal.

Region	Menu	E (kcal)	P (g)	C (g)	S (g)	DF (g)	L (g)	SFA (g)	Na (mg)
	RMV	600 kcal	22.5 g	82.5 g	60 g	7.5 g	20 g	6.7 g	600 mg
**North**	**Less Calorie Menu**								
	First course nº 1	165	5	12	1	2	10	3	312
	Main course nº 5	120	7	6	2	1	8	1	339
	Dessert nº 1	295	11	32	25	2	13	2	90
	**Complete menu 1**	580	23	50	28	5	31	6	741
	**CRV (%)**	97	102	**61**	47	**67**	**155**	90	**124**
	**Higher Caloric Menu**								
	First course nº 2	305	11	27	1	1	17	6	457
	Main course nº 7	312	12	10	0	0	24	5	278
	Dessert nº 6	475	7	42	12	2	31	17	156
	**Complete menu 2**	1092	30	79	13	3	72	28	891
	**CRV (%)**	**182**	**133**	96	**22**	**40**	**360**	**419**	**149**
**Centre**	**Less Calorie Menu**								
	First course nº 1	41	2	4	1	3	1	0	152
	Main course nº 8	74	11	2	1	1	2	0	211
	Dessert nº 4	144	6	22	21	0	4	1	61
	**Complete menu 3**	259	19	28	23	4	7	1	424
	**CRV (%)**	**43**	**84**	**35**	38	**53**	**35**	15	71
	**Higher Caloric Menu**								
	First course nº 6	229	6	26	2	2	11	2	138
	Main course nº 7	263	11	14	0	1	18	4	201
	Dessert nº 2	638	3	16	15	0	63	9	34
	**Complete menu 4**	1130	20	56	17	3	92	15	373
	**CRV (%)**	**188**	**90**	**68**	28	**40**	73	**224**	62
**South**	**Less Calorie Menu**								
	First course nº 5	79	13	2	0	0	2	0	0
	Main course nº 1	121	15	2	0	0	6	1	16
	Dessert nº 3	244	25	37	25	1	7	0	113
	**Complete menu 5**	444	53	41	25	1	15	1	129
	**CRV (%)**	**74**	**236**	**50**	**42**	**13**	**75**	**15**	22
	**Higher Caloric Menu**								
	First course nº 4	204	16	0	0	0	16	2	167
	Main course nº 3	271	28	3	0	0	16	7	486
	Dessert nº 6	448	29	49	35	4	23	2	10
	**Complete menu 6**	923	73	52	35	4	55	11	663
	**CRV (%)**	**154**	**324**	**63**	**58**	**53**	**275**	**164**	111

The least and most energy-dense complete menus were constructed as theoretical combinations of a first course, a main course, and a dessert for each Portuguese region based on the traditional recipes analysed in this study. These menus were developed exclusively for comparative nutritional assessment and do not represent actual portion sizes or real consumption patterns. Energy and nutrient values are expressed per 100 g of recipe. Reference Meal Values (RMVs) represent the target nutrient content for one complete meal and were calculated assuming that a complete meal provides approximately 30% of the daily reference intake. The RMVs were calculated based on the following daily reference values: 2000 kcal for energy, 15% protein, 55% carbohydrates, 30% total fat, 10% free sugars, 10% saturated fatty acids, 25 g dietary fibre, and a daily sodium intake of 2000 mg, corresponding to the safe and adequate intake established by the European Food Safety Authority (EFSA) [14]. Values highlighted in bold are considered nutritionally inadequate. CRV (Comparison with Reference Value) percentages between 90 and 110% were considered adequate for energy and macronutrients. For free sugars, saturated fatty acids, and sodium, values exceeding the reference value were classified as excessive, whereas for dietary fibre, values below 90% of the reference were considered insufficient. Abbreviations: E, energy; P, protein; C, carbohydrates; S, sugars; DF, dietary fibre; L, total lipids; SFA, saturated fatty acids; Na, sodium; CRV, Comparison with Reference Value; RMV, Reference Meal Value.

## Data Availability

The original contributions presented in this study are included in the article. Further inquiries can be directed to the corresponding author(s).
